# A tuff interlayer in deep potash-bearing salt rocks and its implication for potash mineralization in the Simao Basin, southwestern China

**DOI:** 10.1038/s41598-022-20789-1

**Published:** 2022-09-29

**Authors:** Zhong-Ying Miao, Mian-Ping Zheng, Peng-Cheng Lou, Dong Wang, Qi-Hui Xu, Jian-Ming Xu

**Affiliations:** 1 MNR Key Laboratory of Saline Lake Resources and Environments, Institute of Mineral Resources, Chinese Academy of Geological Sciences (CAGS), Beijing, 100037 China; 2 School of Earth Sciences and Resources, China University of Geosciences, Beijing, 100083 China; 3 MNR Key Laboratory of Metallogeny and Mineral Assessment, Institute of Mineral Resources, CAGS, Beijing, 100037 China

**Keywords:** Mineralogy, Petrology, Sedimentology

## Abstract

The lithology and genesis of a dark grey clastic interlayer first encountered within the deepest potassium-rich salt body in the Simao Basin, southwestern China, were analysed. Analyses of the petrography, mineralogy, and element geochemistry of the layer revealed that (1) the layer contains quartz crystals with gulf corrosion edges and explosion cracks and angular volcanic ash-sized glasses; (2) the main mineral components of the crystal fragments are chlorite, illite, biotite, quartz, anhydrite, gypsum, magnesite, pyrite, molybdenite, clinopyroxene, and zircon; (3) the rare earth element patterns, Zr/TiO_2_ and Nb/Y diagrams as well as boron content all indicate a volcanic origin for the layer. Based on these observations, the layer is suggested to be an altered tuff associated with various volcanic fragments dominated by chlorite and formed after alteration of a parent tuff in an alkaline, salty, and low-temperature water body. Discovery of the layer indicates that the potash-bearing salt rocks could have taken in volcanic materials during these volcanic activities and provides the possibility of reliable zircon U‒Pb dating to determine the absolute age of the host rock, which is fundamental in studying the genetic mechanism of this deeply buried salt body.

## Introduction

Evaporite basins are often accompanied by volcanic activities during salt precipitation, and these activities contribute to the salt enrichment of the basins^1,2^. Examples of this basin-volcanic activity ‘partnership’ can be found in many locations around the world, including the Paleogene evaporite Shahejie Formation (Fm.) in the Dongpu Sag, Bohai Bay Basin^1^, the Paleocene evaporite layers in the Jiangling Sag, Jianghan Basin^2^ in East Asia, the giant evaporite Neogene belt in the central Andes^3^ in South America, and the Neogene evaporite basin in western Turkey^4^. However, pyroclastic interlayers formed by codeposition of pyroclastic deposits and evaporites are rare, except for the reported tuff interlayer in the Triassic polyhalite-bearing salt rocks in the Sichuan Basin^5^ and the described basalt-andesitic pyroclastic rock interlayer in the salt rocks of the Mengyejing potash deposit in the Simao Basin^6^. Pyroclastic interlayers of salt rocks indicate the contributions of volcanic sources to the salt deposit and have important chronological significance for constraining the salt deposit model^4,7,8^, so they are important research objects for studies of mineral deposits.

The deepest potash-bearing salt body found to date is buried between 2397.0 and 2650.0 m, as revealed by borehole MK-3 in the Simao Basin, southwestern China. It hosts a lithologically unique interlayer at depths between 2594.4 and 2594.7 m that is lithologically different from the surrounding rocks. However, the layer shares similar characteristics with shallow pyroclastic rocks, as described in previous studies^6^. This paper is intended to confirm the pyroclastic lithology of the layer and discusses its genesis through petrological, mineralogical, and geochemical analyses, thus laying a foundation for further and more accurate dating of metallogenesis and determination of the genesis of the salt body, as well as establishment of a possible genetic relationship between deep and shallow potash deposits.

## Geological setting

The Simao Basin is located in the northern Indochina Block and is bounded by the Jinshajiang-Ailaoshan suture zone to the east and by the Lancangjiang suture zone to the west^9,10^. Influenced by subduction of the Indian Plate to the Eurasian Plate^11^, the formations and main faults in the basin are generally NW‒SE oriented^12^. In the Middle-Late Triassic, the basin had the characteristics of a rift basin^13^ with volcanic rocks developed in the basin margin^14^. In the Jurassic‒Cretaceous, the basin took the shape of an intracontinental depression basin^13^.

The Middle-Upper Triassic layer in the basin contains a variety of marine clastic and carbonate deposits. Overlaying the Upper Triassic are coastal swamp clastic rocks and coal seams with the edge of the basin topped by alkaline, high-aluminium series, and intermediate-basic volcanic rocks^13^. The Lower Jurassic layer mainly hosts fine clastic rocks of tidal flat and lagoon facies. The Middle Jurassic contains barrier beaches and shell beaches in the western part of the basin and tidal flats behind beaches in the eastern part of the basin. The Upper Jurassic rocks are largely continental red fine clastic rocks. The Cretaceous is a set of typical fluvial-lacustrine sandstones, shales, and conglomerates^15^.

To date, only one potash deposit, the Mengyejing potash deposit in the Mengyejing Formation, has been discovered in the Simao Basin. That deposit is buried between 27.0 and 1251.0 m and is a chloride-type potassium salt deposit containing compounds such as NaCl, KCl, KCl·MgCl_2_·6H_2_O, CaSO_4_, and MgCO_3_. With an average KCl content of 8.81%, the deposit hosts 1.676 billion tons of KCl resources. The genetic mechanism of the deposit determined by previous studies is as follows: the deposit was solid salt diapirs that migrated from deep due to tectonic activities^16,17^; the potash deposit was of continental origin with a remnant seawater trace^18^ and was characterized by seawater migration and metamorphism in multistage basins^19^.

Since very little diagnostic evidence has been previously published, the Mengyejing Fm. has yet to be chronostratigraphically determined. Sporopollen assemblage analysis suggested that the formation was between the Aptian and Albian^20^. Based on zircon SHRIMP U-Ph dating, the age of the formation has been constrained between 100 and 110 Ma (from Albian to Cenomanian)^21^. The first proposed age sequence of > 112 Ma to ca. 63 Ma for the formation^22^ was obtained through detrital zircon U‒Pb geochronologic-magnetostratigraphic dating.

Available data show that the Middle Jurassic Hepingxiang Fm. is another stratum worth exploring for potash deposits, and its salt-bearing layers are most likely to be deposited in a salt sag on a tidal flat (i.e., the intertidal depression or supratidal salt marsh)^23^.

## Samples

### Samples

**Figure 1:**
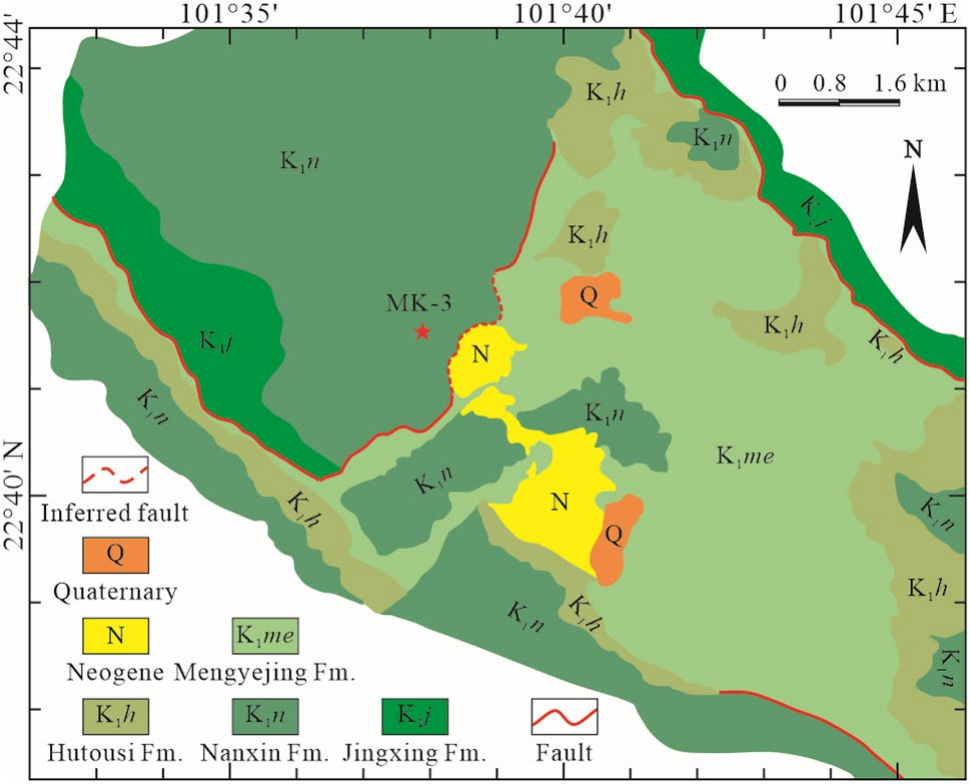
Geological map of the Mengyejing potash mining area and the location of well MK-3. In the map, the locations of the potash deposit, the MK-3 well and the distributions of faults and strata are shown.

**Figure 2:**
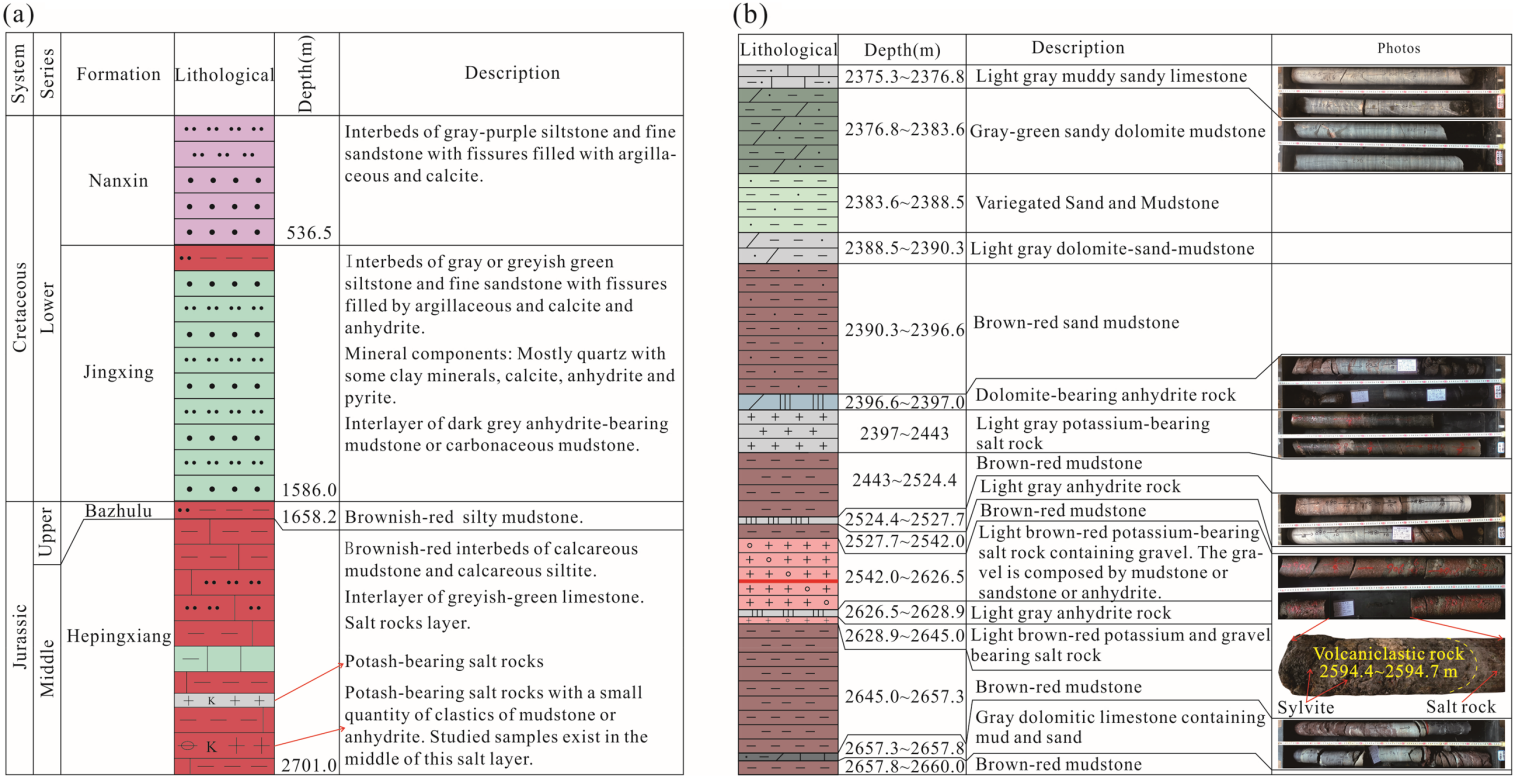
Stratigraphic characteristics. (**a**) Stratigraphic sequence penetrated by borehole MK-3. (**b**) Lithology column and sampling locations in the salt-bearing section of well MK-3.

From June 2018 to June 2019, the Institute of Mineral Resources under the Chinese Academy of Geological Sciences hosted a geological survey of a potash deposit in areas outside the Mengyejing potash mining area, Jiangcheng Depression, Simao Basin, using an exploratory well with a designed drilling depth of 2700.0 m. The project was jointly funded by the Geological Survey Project (DD20160054) and Potash Deep Field Special Project (2017YFC0602801). Exploratory well MK-3 was drilled at the coordinates 101°37′43.5" E and 22°41′ 25.0" N, where outcropped strata were observed to be the Lower Cretaceous Nanxin Fm. (Fig. 1). Figure 2a shows the stratigraphic sequence of the Nanxin Fm., Jingxing Fm., Bazhulu Fm., Hepingxiang Fm. (from top to bottom) penetrated by borehole MK-3.

The well was drilled through two layers of evaporite rock with a cumulative thickness of 149.0 m, and one layer was buried between 2397.0 and 2443.0 m and the other between 2542.0 and 2645.0 m. At depths between 2594.4 and 2594.7 m in the latter layer, a 30-cm-thick layer of dark grey "clastic rock" was found to be sandwiched between potash-bearing salt rocks and showed a significantly larger diameter after it was brought up to the surface and removed from the coring tube. On-site checking showed that the "clastic rocks" had barely consolidated, and were covered with fissures filled with orange‒red sylvite (Fig. 2b). The main mineral components included quartz, anhydrite, clay minerals, and sylvite. The focus of this study, as shown in Fig. 2, is these pyroclastic rocks, particularly the sample labelled MK-3-T.

### Contact relationship between the layer and salt rocks

The sample analysed in this paper was from the lower part of the core gathered during the 906th coring trip into well MK-3. The trip started at a depth of 2591.9 m and ended at a depth of 2594.9 m. The interlayer (in situ sediment) with a length of 0.30 m was found to be sandwiched between two salt rocks. The top was from mud or gypsum-bearing salt rocks and potash-bearing salt rocks with a length of 2.5 m (from 2591.9 to 2594.4 m), and the bottom was mud or gypsum-bearing rocks with a length of 0.2 m.

Well MK-3 was protected by a 146 mm inner intermediate casing from the surface to a depth of 1900.0 m. The well passed successively through red, brown‒red, and purple‒grey siltstone intercalated with 5 to 15 cm thick grey‒green mudstone from 1900.0 to 2397.0 m, dark grey potash-bearing salt rock from 2397.0 to 2443.0 m, brown‒red silty mudstone from 2443.0 to 2535.0 m, black carbon mudstone from 2535.0 to 2542.0 m, and mud-bearing salt rock or gypsum-bearing salt rocks from 2542.0 to 2595.0 m. All of these lithologies were quite different from the samples, which means there was no possibility that these samples were from fragments or blocks that fell from the formation.

After being brought up to the surface, the sample expanded due to the pressure release, making the contact between the sample and the overlying salt layers difficult to identify. However, the contact between the sample and the underlying salt layer was clear (Fig. 2b), and it served as additional evidence that the sample was in situ sediment from a sedimentological point of view.

In conclusion, drilling engineering, petrology, and sedimentology showed that the sample was a “clastic rock” deposited in situ between salt layers, laying a solid foundation for further exploration of its geological significance through sedimentological, petrological, and mineralogical analyses.

## Results

### Petrography

**Figure 3:**
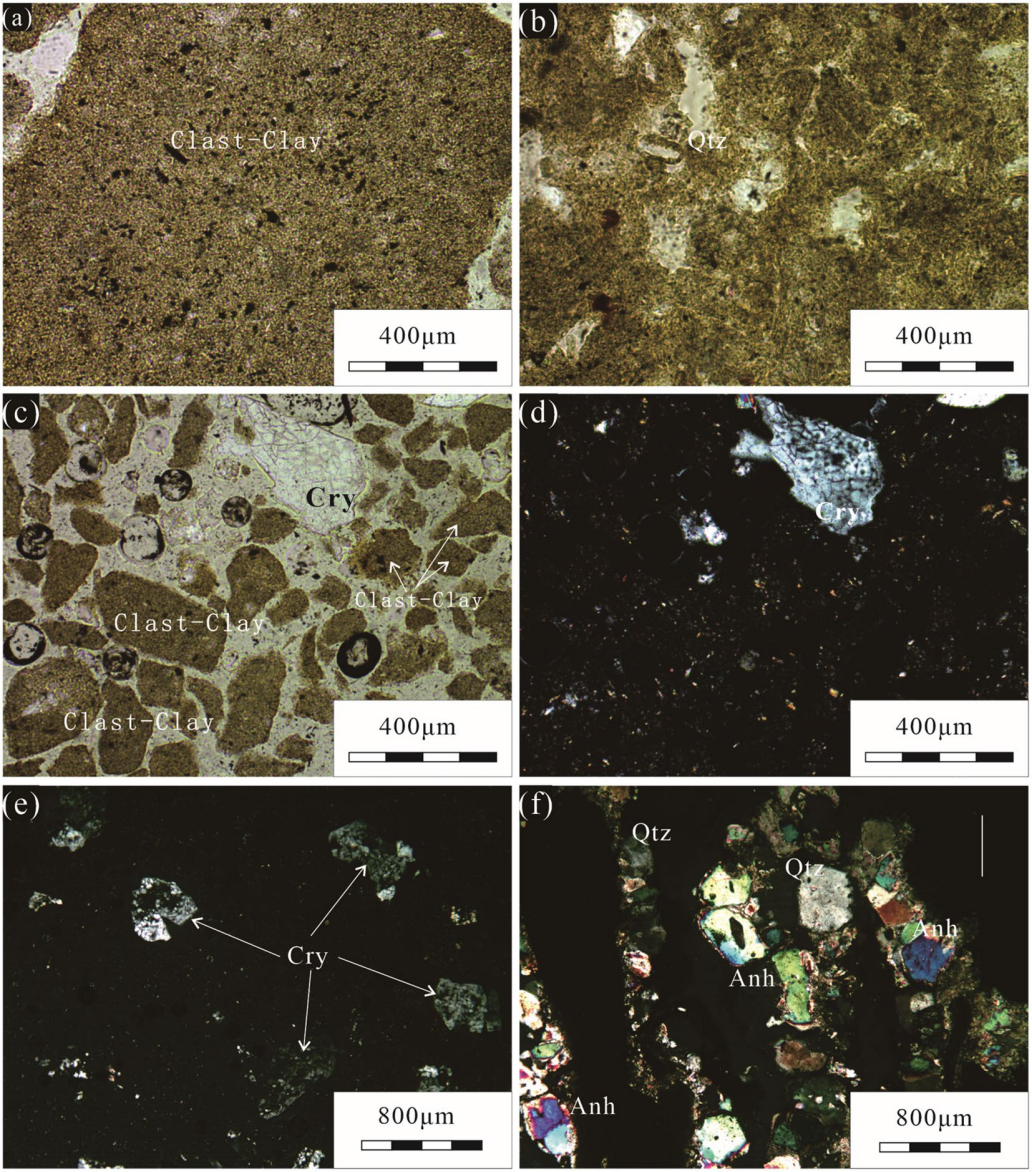
Photomicrographs showing typical characteristics of sample MK-3-T under polarized light; (**a**) very small grains, (**b**) monocrystalline α-quartz, (**c**–**e**) crystal fragment with irregular cracks, (**f**) crystals with larger-sized grains (*Cry* crystal fragment, *Qtz* quartz, *Anh* anhydrite).

The sample was not well consolidated and contained cracks 0.1 to 2 mm wide filled with orange‒red sylvite (Fig. 2b). Under a polarizing microscope, the sample was seen to comprise very small particles with undistinguishable single minerals (Fig. 3a). Alpha-quartz (ca. 120 μm in size) with a perfect crystalline structure was found occasionally in the argillaceous bottom (Fig. 3b). The clay minerals were mostly 0.1 to 1.2 mm clastic particles (Clast-Clay) (Fig. 3a,c), among which 0.4 to 0.8 mm-sized crystal fragments with irregular cracks were observed (Fig. 3c‒e); the sample had extremely low textural maturity, with 0.3 to 0.4 mm-sized quartz and 0.1 to 0.4 mm-sized anhydrite particles occasionally gathered and distributed among the debris particles bonded by clay (Fig. 3f).

### Mineralogy

**Figure 4:**
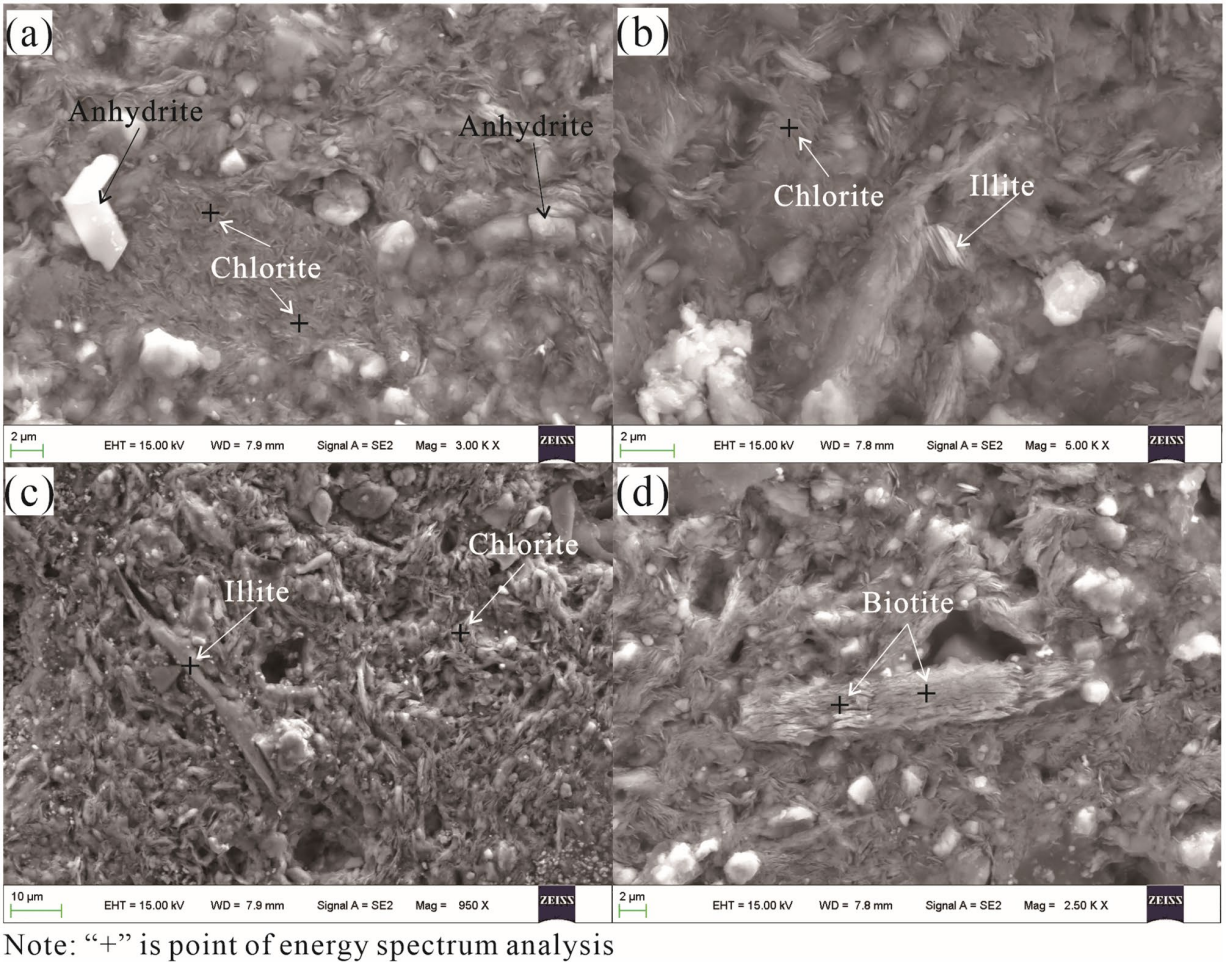
SEM images showing textural features of clay minerals. (**a**) Dominant chlorite and (**b**) flaky petaloid textures, (**c**) illite size and crystal structure and (**d**) close-up of biotite displaying the crystal size and texture.

Based on SEM analyses, the main mineral components of the analysed samples were clay minerals, quartz, gypsum, anhydrite, biotite, pyrite, zircon, clinopyroxene, monazite, molybdenite, and magnesite. Clay minerals dominated and were largely composed of chlorite, illite, and biotite. The chlorite crystal grains were fine and generally ranged in size from 2 to 5 μm, and they were flaky with aggregates of scale and petal shapes (Fig. 4a,b). Flaky illite crystals with various sizes ranging from 2 to 16 μm were dispersed in the chlorite, and some individual flakes had developed threadlike edges pointing towards pores among the mineral grains. Aggregated illites are granular and thin and may be the result of feldspar alterations or chlorite transformations (Fig. 4b,c). Biotites were rarely seen, but aggregates of biotites were occasionally spotted, with cross-sections measuring approximately 4 × 27 μm (Fig. 4d).

**Figure 5:**
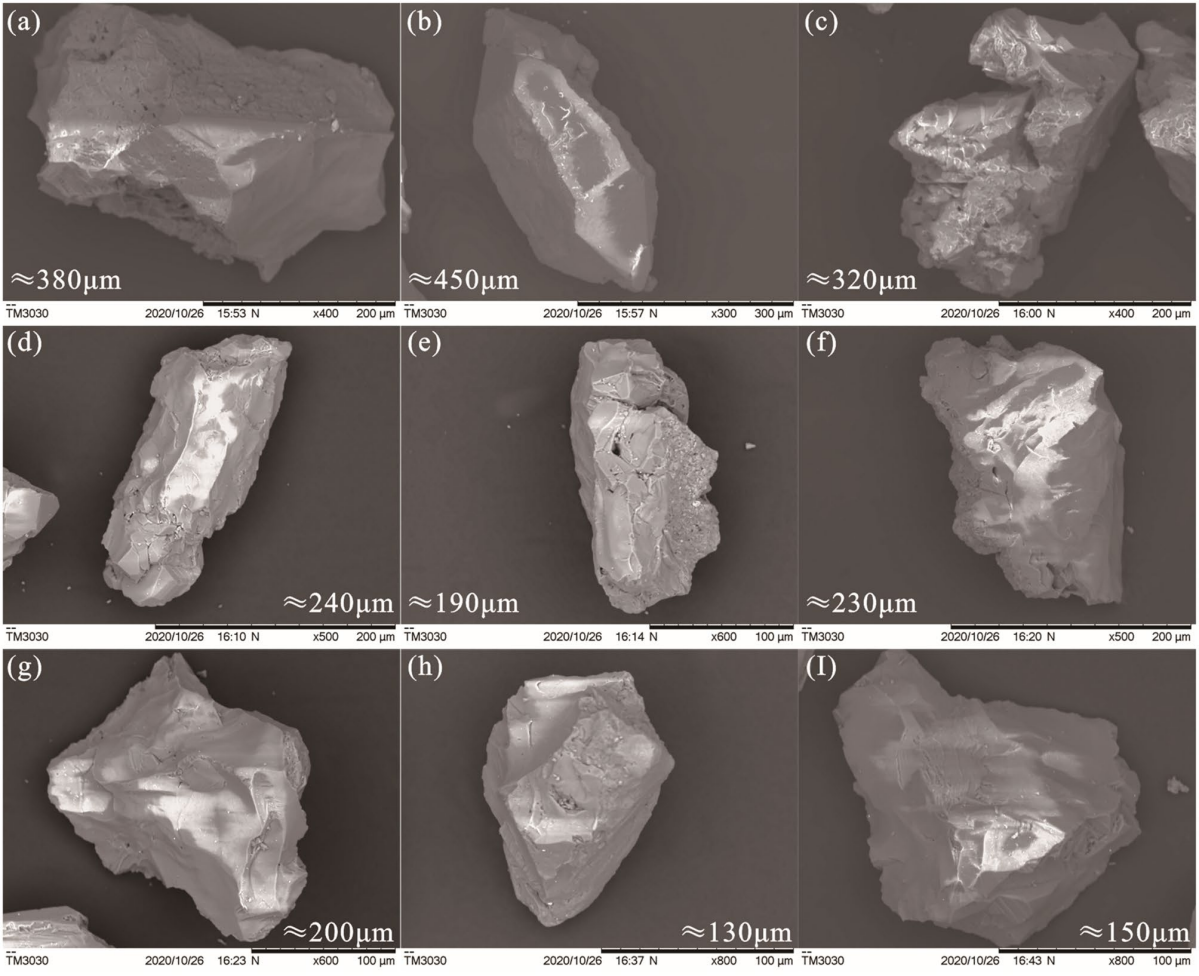
SEM images of quartz particles in sample MK-3-T. The sizes of the quartz, gulf corrosion, and cracks are shown.

**Figure 6:**
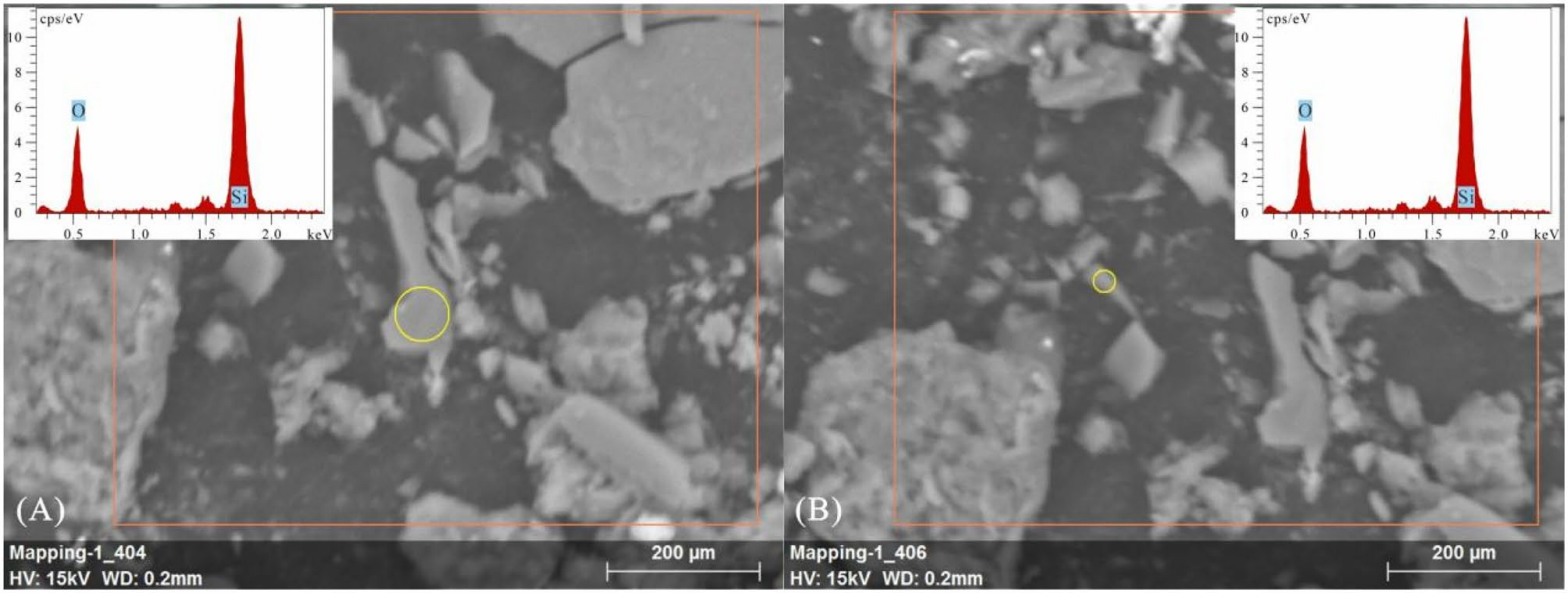
SEM image and EDS data for volcanic glass in the sample.

The sample also contained many quartz particles, which were only located next to clay minerals. The quartz particles or crystals, largely measuring 130 to 145 μm in size, were mostly angular and poorly rounded (Fig. 5). A few cube-shaped crystals with cylindrical and pyramidal faces were observed. Some crystals showed gulf corrosion (Fig. 5b,d,f,g) and cracks (Fig. 5c,e). Volcanic glass with the same chemical composition as the quartz crystals was also present; the largest pieces were as large as approximately 220 μm (Fig. 6A), and the smallest were less than 100 μm (Fig. 6B); according to EDS analysis, they comprised mainly Si and O.

**Figure 7:**
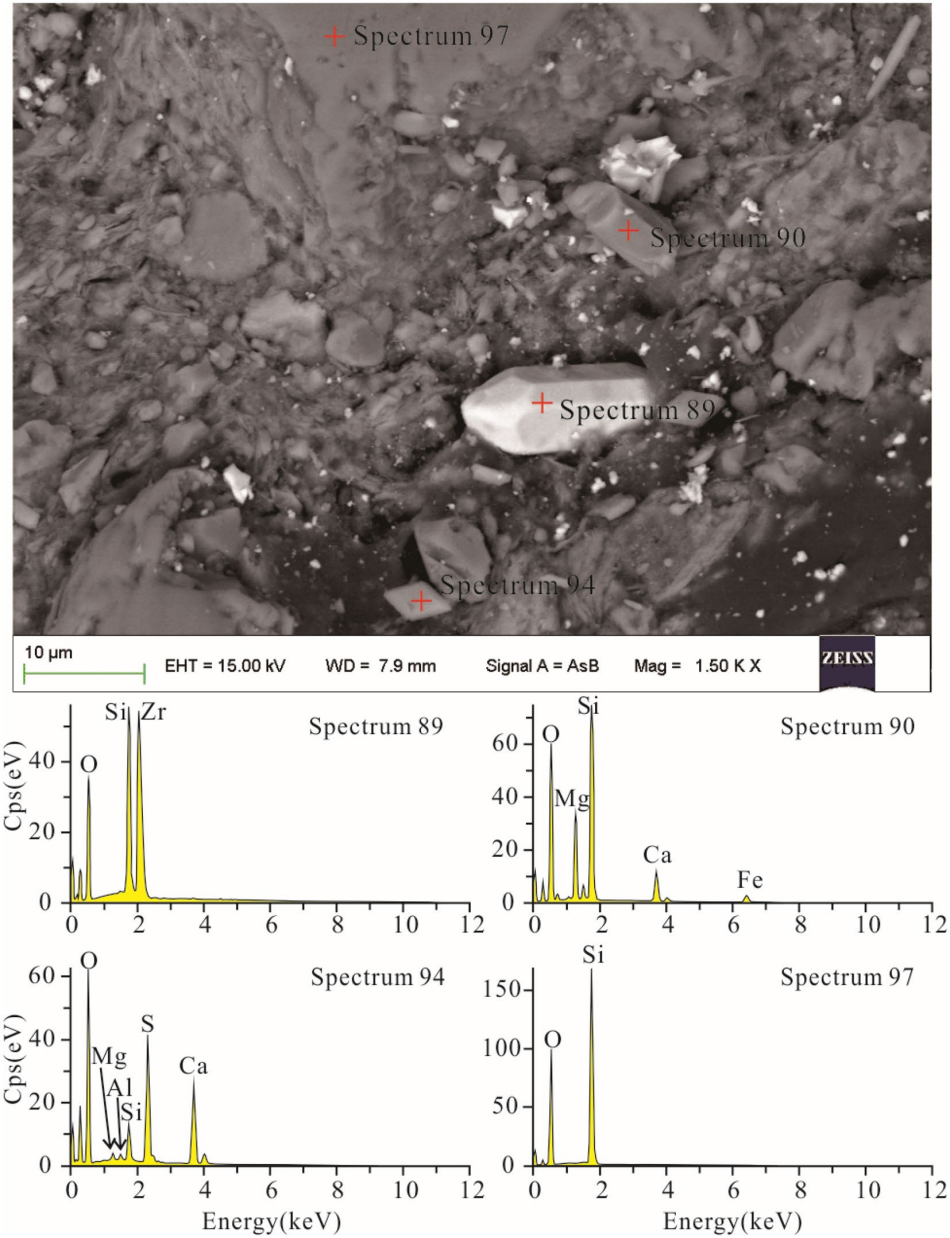
Electron microscope image and energy spectra of zircon, gypsum, quartz, and clinopyroxene in sample MK-3-T.

Gypsum and anhydrite were abundant in the sample, although they were not as abundant as the clay minerals or quartz. Their EDS results revealed an elemental composition consisting primarily of S, O, and Ca, and the chemical composition was shown to be CaSO_4_. The needle-shaped or long columns were gypsum particles, and the diamond-shaped and near cubic ones were anhydrite crystals, as shown by SEM (Fig. 7-Spectrum 90).

Scattered among the clay minerals were monazite, pyrite, molybdenite, magnesite, zircon, and clinopyroxene. The monazite and molybdenite grains were rather small, only approximately 10 to 20 μm in size, and they had poorly formed crystals. The magnesite grains were approximately 25-μm crystals that were well-formed and contained some cracks. The pyrite crystals were relatively large, approximately 100 μm in size and had perfect diamond shapes.

Zircon was associated with clinopyroxene (Fig. 7). The zircon crystals were mostly a combination of double prisms and bipyramids, as shown by SEM, similar to those formed in alkaline rocks or meta-alkalescent granite, and the EDS results showed that the main elements were Si, Zr, and O (Fig. 7-Spectrum 89). Their sizes were ca. 8 × 20 μm. The clinopyroxene crystals were also short and columnar with sizes of approximately 3 × 10 μm.

### Geochemistry

The total REE content of the sample was 141.9 μg/g, which was significantly higher than those of basalts in Jingdong and Mojiang (within the basin) but significantly lower than those of granite from Lincang at the basin margin and Post Archean Australian Shale (PAAS) (see Supplementary Table S1). The sample contained more LREEs than HREEs, with an LREE/HREE ratio of 8.35 (see Supplementary Table S2), which was similar to those of Lincang granite and PAAS and significantly higher than those of the Mojiang basalts and Jingdong basalts.

**Figure 8:**
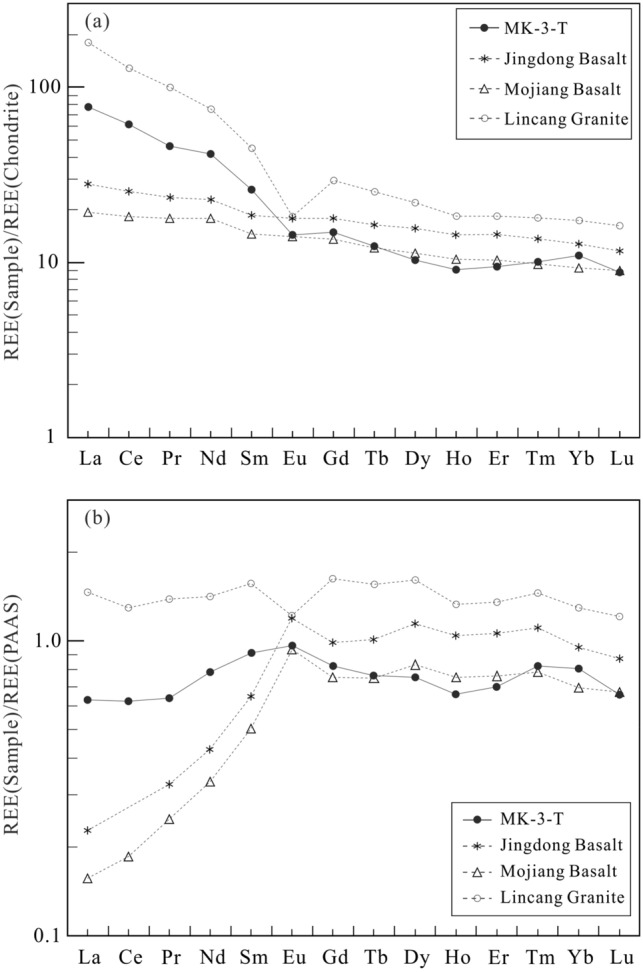
Distribution patterns for REEs in sample MK-3-T, basalt from Jingdong^24^ and Mojiang^25^ and granite from Lincang^25^ (Chondrite date according to reference^26^; PASS date according to reference^27^). The REE distribution patterns of the sample are between those of acidic igneous rocks and basic igneous rocks in the research area.

The chondrite-normalized REE patterns of the sample as well as of basalts in Jingdong and Mojiang and granite in Lincang are shown in Fig. 8a. The patterns were LREE enriched, with (La/Yb)_N_ and (La/Sm)_N_ values of 7.19 and 2.97, respectively, which are both higher than those of basalts from Jingdong and Mojiang but lower than those of granite from Lincang and PAAS, indicating relatively weak LREE differentiation. The (Gd/Yb)_N_ of the sample was 1.39, similar to those of basalts from Jingdong, Mojiang and PAAS, but significantly lower than that of Lincang granite, indicating weak HREE differentiation. The sample had a *δ*Ce_1_ at 1.0, similar to those of chondrites, PAAS, and Jingdong-Mojiang basalts, but slightly higher than that of Lincang granite. It had a *δ*Eu_1_ at 0.70, similar to that of PAAS, but significantly higher than that of Lincang granite and lower than those of Jingdong and Mojiang basalts.

Figure 8b shows the PAAS-normalized REE patterns. The patterns revealed that (1) REE proportions in the sample were generally lower than those in PAAS, and their ratios to the corresponding elements in PAAS were between 0.62 and 0.96; (2) the sample was relatively rich in MREEs (Sm, Eu, and Gd) and HREEs (Er, Tm, and Yb), resulting in an M-shaped distribution curve; (3) it differed from Lincang granite in that the sample had a relatively flat PAAS-normalized curve while that for granite was above that for the PAAS (indicating a higher content of REEs than that of PAAS) and with an apparent negative Eu anomaly; and (4) the pattern of the sample was also different from those of Jingdong and Mojiang basalts, whose PAAS-normalized patterns with inverted “L” shapes indicated lower LREEs and higher MREEs (Eu) than PAAS, and a similar HREE to PAAS.

## Discussion

### Rock genesis revealed by mineral assemblage characteristics

Mineral assemblage characterization was performed to reveal the lithology of the sample studied. Polarizing microscope observations showed that the sample mainly contained clay minerals and small amounts of anhydrite, quartz, and crystal fragments with irregular cracks, which are typically found in debris from volcanic eruptions^28^. The analysed pyroclastic rocks contained volcanic glass fragments, crystal fragments, and quartz (with gulf erosion), which were also observed through SEM. Mineral assemblages such as pyrite, clinopyroxene, and molybdenite were observed, and their existence generally indicates a supply of hydrothermal fluid minerals from deep volcanic activities^29–31^. Therefore, all of these results suggest that the lithology of the sample was pyroclastic.

SEM also revealed typical evaporite minerals, such as magnesite, anhydrite, and gypsum, scattered throughout the sample. Since the cracks found in clastic rocks were filled with orange‒red potash salt, it is possible that the pyroclastic material was codeposited with saline minerals in an evaporite basin.

SEM showed that the clay minerals were mainly chlorite, with small amounts of illite and mica. This differed from other clastic rocks in the study area^32,33^, thus serving as additional evidence for the pyroclastic identity of the sample.

Based on the combined characteristics of clastic minerals, clay minerals, and evaporite minerals, the pyroclastic rock was possibly deposited synthetically with evaporite rocks. According to the particle size characteristics of pyroclastics^34^, the sample can be defined as tuff. However, due to the special preservation conditions (i.e., wrapped within salt rocks), the tuff was altered but not well consolidated.

### Genesis of chlorite

Chlorite is one of the Fe- and Mg-rich aluminosilicate clay minerals and has a generalized chemical formula of (X, Y)_4–6_(Si, Al)_4_O_10_(OH, O)_8_, in which X and Y represent divalent or trivalent ions, including Fe^2+^, Fe^3+^, Mg^2+^, or Al^3+^ ^35^. Chlorite in sedimentary rocks is formed as a diagenesis product from the transition of detrital Fe-rich berthierine and Mg-rich smectite or from the reaction of kaolinite with Fe and the breakdown of volcanic grains^35–37^. Plagioclase-, pyroxene-, hornblende-, biotite-, Fe- and Mg-rich mafic minerals commonly seen in igneous rocks can also be converted into chlorite by hydrothermal alteration^35,38^.

Volcanic rock fragments have long been proposed as a way of delivering the key ingredients for formation of authigenic chlorite^36^. Glassy components in tuffs can be devitrified and form smectite, which can then react with iron oxide and saline water to produce chlorite^39^ if the environment is alkaline (pH ca. 8), has a temperature of 30 − 55 °C and a shallow water depth^40^. Ca^2+^ and Na^+^ are removed from montmorillonite layers, and Fe^2+^ and Mg^2+^ in the environmental fluid are combined with OH^-^ from the aqueous solution to form the brucite-like layer, which enters the montmorillonite layer in the form of octahedral sheets and combines with the original montmorillonite structure to form a 2:1:1 chlorite^41^.

The Simao Basin was within a hot subtropical zone near 29° N during the Mesozoic^42,43^. Research on the homogenization temperatures of salt rock fluid inclusions has shown that the paleotemperature during the salt evolution period was between 35 and 65 ℃^44^. The magnesite found in the present sample indicates a slightly alkaline sedimentary environment. A relatively high salinity water body can be inferred from the presence of potash-bearing salt rocks that wrapped the sampling layer. Volcanic activity may serve as a carrier of Fe^2+^ and Mg^2+^ into the environmental fluid. As a result, the study area had ideal temperature, salinity, pH, and provenance conditions for alteration of tuff to chlorite. To be more specific, glass fragments in the tuff were devitrified into montmorillonite, which was then chloritized in an Fe- and Mg-rich environment. While the main body underwent chloritization, individual crystals were illitized because of the presence of K^+^ in the environment.

### Geochemistry

REEs in sedimentary rocks are mainly controlled by provenances and less by diagenesis and alteration and therefore can be used to reveal the characteristics of REEs in parent rocks^27,45,46^.

**Figure 9:**
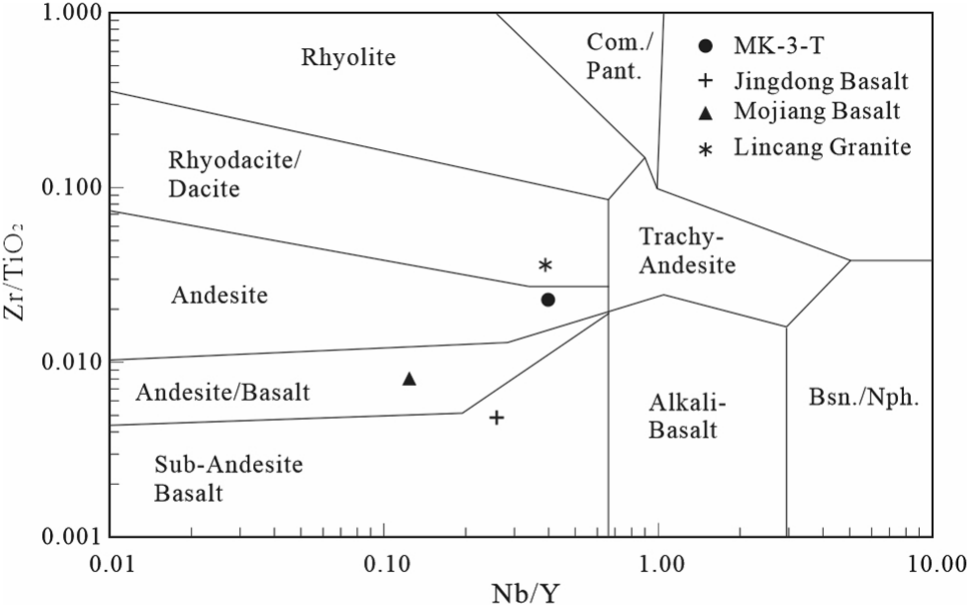
Zr/TiO_2_ vs. Nb/Y shows typical volcanic rock types (modified after reference^51^). The original lithology of the sample is andesite tuff (*Com* comendite, *Pant* pantellerite, *Bsn* basanite, *Nph* nephelinite).

The chondrite-normalized and PAAS-normalized REE distribution patterns in the sample were somewhere between those of Lincang granite on the basin edge and those of Jingdong and Mojiang basalts within the basin, indicating an intermediate volcanic rock origin. Previous studies also showed that acidic rocks have obvious negative *δ*Eu anomalies^25,47,48^, and basic rocks have weak negative or positive *δ*Eu anomalies^24,49,50^, both at the edge of and inside the Simao Basin. The analysed sample had *δ*Eu values between those of the acidic rocks (0.20–0.38) and basic rocks (0.85–1.09), which is additional evidence showing that the sample has the characteristics of an intermediate rock. The Zr/TiO_2_ and Nb/Y diagrams of the elements further identified the parent source of the sample as andesite with typical extrusive rock characteristics (Fig. 9).

Boron content can also indicate the volcanic origin of a sample. The present sample has a boron content of 580 μg/g, which is abnormally high compared with salt and clastic rocks from the Mengyejing area^52^ (see Supplementary Table S3). Some researchers have suggested that this is likely caused by the introduction of hydrothermal fluids or hot springs through volcanic activities^53,54^. This also supports the proposal that the sample is a clastic deposit formed by volcanic eruptions.

Parameters derived from REE ratios such as *δ*Ce are generally used to reflect the redox potential of the sedimentary environment^55–57^. In an oxidizing setting, the Ce in water is more likely to migrate and settle in underwater sediments, resulting in an accumulation of Ce in clastic sediments that presents as positive *δ*Ce anomalies, whereas in a reducing environment, Ce is unlikely to migrate to underwater sediments and cause *δ*Ce negative anomalies^58^. The *δ*Ce_1_ and *δ*Ce_2_ values of the samples were 1.00 and 1.03, respectively, and showed no obvious *δ*Ce-related anomalies; this reflects weakly oxidizing‒reducing conditions. This was further confirmed by the mineral assemblages in the sample, where anhydrite coexisted with pyrite, molybdenite and other sulfides.

### Genetic model and geological significance

Based on the above analyses, the genesis of this intersalt tuff may be described as follows: when so concentrated as to precipitate sylvite, the brine water body in this evaporite basin of Simao shrank a great deal but still maintained a redox interface, below which a (at least) 15 cm high accommodation space can be inferred from the thickness of the sample. The basin was supplied from time to time with terrigenous freshwater, as the footprint was recorded by terrigenous clastic materials commonly seen in evaporite salt rocks in the basin. Volcanic ash from volcanic eruptions was sent to float over the basin, where it was drawn down by gravity and deposited as pyroclastic rocks near the redox interface. The rocks were then subjected to an alkaline, hyperhaline, low-temperature, and aqueous environment, with volcanic glasses first turning into montmorillonite and then to chlorite with a small amount of illite. As the alterations proceeded, newly formed potash-bearing evaporite rocks overlaid and wrapped these pyroclastic rocks together with underlying potash-bearing evaporite layers. During the process, water adsorbed by clay minerals was also sealed within and had been keeping these fragmented rocks from final diagenesis even with burial depths up to ca. 2600.0 m, formation temperatures ranging between 96.2 and 96.6 ℃ and period of more than 100 Ma long. This may explain the coexistence of gypsum and anhydrite as well as the abnormally high loss (28.01%) on ignition for major elements in the sample.

The material sources of the evaporites in the Simao Basin are still under debate. Some suggest seawater as the primary source^19^, and some propose terrestrial water^18,59^. The anhydrite sulfur isotopes in these evaporite rocks indicate that volcanic activity had an important impact on the material supply for the evaporite rocks^60^. Analyses of hydrochemical characteristics of spring water and the genesis of surface potassium anomalies allow us to conclude that deep volcanic rocks were one of the important material sources of the evaporite rather than volcanic activity occurring during evaporite precipitation^61^.

This study provides direct evidence of volcanic activity occurring during the salt rock deposition period. The pyroclastic layer itself is chronologically significant and allows more accurate dating of potash salt deposits. Furthermore, since no evidence thus far has indicated that volcanic eruptions occurred inside the Simao Basin during the Jurassic‒Cretaceous (indicating a relatively stable structure and deposition of the basin during the period), these pyroclastic rocks are very likely to be the result of structural movements and volcanic activities triggered by the Tethys closure in the periphery of the basin.

## Conclusion

Petrography, mineralogy and element geochemistry all indicate that the studied sample is tuff. A reasonable inference for the genesis of the tuff is that (1) during the precipitation of potash-bearing salt, a volcanic eruption that was probably triggered by the closure of the Meso-Tethys occurred outside the Simao Basin; (2) tephra ejected from the volcanic eruption eventually was deposited in the evaporite and was later altered in an alkaline, hyperhaline, and low temperature water environment to form a tuff layer; and (3) the tuff layer covered by potash-bearing salt rocks was then buried with it under clastic rocks deposited later .

The discovery of the tuff layer not only suggests a volcanic material supply for the potash-bearing salt rocks but also provides an opportunity to perform reliable zircon dating to determine the absolute age (of the tuff), which could then be used to explore the genesis of the salt rocks.

## Methods

### Transmitted light microscopy

Transmitted light microscopy was performed on samples after fixing them on a glass slide using epoxy resin as adhesive and grinding the sample to a thickness of 0.03 mm with saturated saline; a Leica 2700P polarizing microscope with a LED light source and a LAS imaging system was used for observations.

### Scanning electron microscopy (SEM)

A Zeiss Ultra Plus Field Emission Scanning Electron Microscope (FESEM) with an accelerating potential of 15 kV was used. The mineral components of the samples were determined with an Oxford X-MaxN80 dual-detector EDS (X-ray Energy Dispersive Spectrometer).

Some debris adhered to the sample tray and was tested with a TM3030 scanning electron microscope and an XFlash MINSVE energy spectrometer.

### Rare earth element analyses

The rare earth element (REE) contents of the samples were determined with an ELEMENT XR Inductively Coupled Plasma Mass Spectrometer (ICP‒MS) operated at an ambient temperature of 23.5 °C and relative humidity of 41%.

The analytical procedure consisted of weighing 25 mg (up to 0.01 mg) of the powdered sample (< 74 µm in size), putting it into a Teflon vessel insert, and then dissolving it with approximately 1 mL of HF (1.16 g/mL) and approximately 0.5 mL of nitric acid (1.42 g/mL). The insert was sealed, put into an oven and heated to 185 ± 5 °C for 24 h. The insert was removed from the oven, cooled and heated nearly to dryness on an electric hot plate. Nitric acid (0.5 mL, 1.42 g/mL) was added to the tank and allowed to evaporate until dry. This step was repeated after 5 mL of nitric acid (1 + 1) was added to the insert and sealed. The samples were placed into the oven and heated at 130 °C for 3 h. The insert was removed from the oven, and the solution was transferred to a 25 mL plastic bottle and diluted to 25 mL with deionized water; it was shaken and prepared for ICP‒MS measurements.

## Supplementary Information

Supplementary Information.

## Data Availability

Please contact Dr. Zhong-Ying Miao by e-mail (zhymiao@foxmail.com), if someone wants to request the data from this study.
