# Supplementary material for: A tuff interlayer in deep potash-bearing salt rocks and its implication for potash mineralization in the Simao Basin, southwestern China
Source: Sci Rep. 2022 Sep 29;12:16320. doi: 10.1038/s41598-022-20789-1 (PMC9522788; doi:10.1038/s41598-022-20789-1)
Supplement: Supplementary file 1 — Supplementary Information. [file 41598_2022_20789_MOESM1_ESM.docx]

**Supplementary table 1**: Relative contents of REEs in sample MK-3-T.

| Sample No. | LREE (μg/g) | | | | | | HREE (μg/g) | | | | | | | | |
| --- | --- | --- | --- | --- | --- | --- | --- | --- | --- | --- | --- | --- | --- | --- | --- |
|  | La | Ce | Pr | Nd | Sm | Eu | Gd | Tb | Dy | Ho | Er | Tm | Yb | Lu | Y |
| MK-3-T | 24.00 | 49.90 | 5.62 | 25.10 | 5.09 | 1.06 | 3.87 | 0.59 | 3.31 | 0.65 | 1.99 | 0.33 | 2.25 | 0.28 | 17.90 |
| Jingdong Basalt^1^ | 8.66 | 20.53 | 2.87 | 13.68 | 3.63 | 1.31 | 4.63 | 0.78 | 5.04 | 1.03 | 3.03 | 0.44 | 2.67 | 0.38 | 33.69 |
| Mojiang Basalt^2^ | 5.97 | 14.81 | 2.19 | 10.69 | 2.82 | 1.03 | 3.53 | 0.57 | 3.65 | 0.75 | 2.17 | 0.31 | 1.94 | 0.29 | 23.49 |
| Lincang Granite^2^ | 55.59 | 103.76 | 12.17 | 45.28 | 8.73 | 1.33 | 7.64 | 1.20 | 7.06 | 1.32 | 3.86 | 0.58 | 3.62 | 0.52 | 38.40 |
| Chondrite^3^ | 0.31 | 0.81 | 0.12 | 0.60 | 0.20 | 0.07 | 0.26 | 0.05 | 0.32 | 0.07 | 0.21 | 0.03 | 0.21 | 0.03 | 1.96 |
| PAAS^4^ | 38.00 | 80.00 | 8.80 | 32.00 | 5.60 | 1.10 | 4.70 | 0.77 | 4.40 | 0.99 | 2.85 | 0.40 | 2.80 | 0.43 | 27.00 |

**Supplementary table 2**: Characteristic parameters of REE in sample MK-3-T.

| Sample No. | ∑REE^(a)^ | ∑LREE^(b)^ | ∑HREE^(c)^ | LREE/HREE^(d)^ | (La/Yb)_N_^(e)^ | (La/Sm)_N_ | (Gd/Yb)_N_ | *δ*Eu_1_^(f)^ | *δ*Eu_2_^(g)^ | *δ*Ce_1_^(h)^ | *δ*Ce_2_^(i)^ |
| --- | --- | --- | --- | --- | --- | --- | --- | --- | --- | --- | --- |
| MK-3-T | 141.94 | 110.77 | 31.17 | 8.35 | 7.19 | 2.97 | 1.39 | 0.70 | 0.73 | 1.00 | 1.03 |
| Jingdong Basalt^1^ | 102.35 | 50.67 | 17.99 | 2.82 | 2.19 | 1.50 | 1.40 | 0.98 | 0.98 | 0.99 | 0.99 |
| Mojiang Basalt^2^ | 74.22 | 37.51 | 13.22 | 2.84 | 2.07 | 1.33 | 1.47 | 1.00 | 1.00 | 0.99 | 0.99 |
| Lincang Granite^2^ | 291.04 | 226.85 | 25.79 | 8.80 | 10.35 | 4.01 | 1.70 | 0.49 | 0.50 | 0.92 | 0.96 |
| Chondrite^3^ | 5.25 | 2.11 | 3.14 | 1.78 | 1.00 | 1.00 | 1.00 | 1.00 | 1.00 | 1.00 | 1.00 |
| PAAS^4^ | 209.84 | 165.50 | 44.34 | 9.54 | 9.15 | 4.27 | 1.35 | 0.64 | 0.66 | 1.02 | 1.05 |

^(a)^∑REE: La‒Lu+Y; ^(b)^∑LREE: La‒Eu; ^(c)^∑HREE: Gd‒Lu; ^(d)^LREE/HREE=∑LREE/∑HREE; ^(e)^(La/Yb)_N_=La_N_/Yb_N,_ corner mark “N” is chondrite-normalized; ^(f)^*δ*Eu_1_=2*Eu_N_/(Sm_N_+Gd_N_); ^(g)^*δ*Eu_2_=Eu_N_/(Sm_N_×Gd_N_)^1/2^; ^(h)^*δ*Ce_1_=2*Ce_N_/(La_N_+Pr_N_); ^(i)^*δ*Ce_2_=Ce_N_/(La_N_×Pr_N_)^1/2^.

**Supplementary table 3**: Boron contents in Mengyejing potash deposit, Simao Basin (from reference 5).

| Lithology | Sample number | B content (ppm) | |
| --- | --- | --- | --- |
|  |  | Range | Average |
| Sylvite rock | 95 | 0~472 | 96.8 |
| Halite rock | 176 | 0~250 | 57.3 |
| Clastic rock | 15 | 97~260 | 144 |
| Anhydrite | 7 | 13~300 | 89 |

**Supplementary references**:

1. Dong, Y.P., Zhu, B.Q., Chang, X.Y. & Deng, S.X. Geochemistry of the two–type volcanic rocks from Ailaoshan suture zone and their tectonic implication. Geochim. 29, 6–13 (2000).

2. Kong, H.L. et al. 2012, Petrogenesis of Lincang granites in Sanjiang area of western Yunnan Province: Constraints from geochemistry, zircon U–Pb geochronology and Hf isotope. Acta Petrol. Sin. 28, 1438–1452 (2012)

3. Boynton, W.V. Cosmochemistry of the rare earth elements: Meteorite studies. Dev. Geochem. 2, 63–114 (1984)

4. Mclennan, S.M. Rare earth elements in sedimentary rocks: influence of provenance and sedimentary processes. Rev. Mineral. Geochem. 21, 169–200 (1989).

5. Xu, X.S. & Wu, J.L. Potash deposits in Mengyejing, Yunnan: A study of certain characteristics, geochemistry of trace elements and genesis of the deposits. Acta Geosci. Sin. 5, 17–36 (1983).
